# The importance of rectal cancer MRI protocols on iInterpretation accuracy

**DOI:** 10.1186/1477-7819-6-89

**Published:** 2008-08-20

**Authors:** Chikako Suzuki, Michael R Torkzad, Soichi Tanaka, Gabriella Palmer, Johan Lindholm, Torbjörn Holm, Lennart Blomqvist

**Affiliations:** 1 Department of Diagnostic Radiology, Institution for Molecular Medicine and Surgery, Karolinska University Hospital Solna and Karolinska Institute, Stockholm, Sweden; 2 Department of Radiology, Uppsala University Hospital, Uppsala, Sweden; 3 Dept. of Oncology, Radiology and Clinical Immunology Section of Radiology Uppsala University Hospital and Karolinska Institute, Uppsala, Sweden; 4 Department of Surgery, Institution for Molecular Medicine and Surgery, Karolinska University Hospital Solna and Karolinska Institute, Stockholm, Sweden; 5 Department of Pathology, Karolinska University Hospital Solna and Karolinska Institute, Stockholm, Sweden; 6 Department of radiology, Danderyd Hospital, Stockholm, and Karolinska Institute, Stockholm, Sweden

## Abstract

**Background:**

Magnetic resonance imaging (MRI) is used for preoperative local staging in patients with rectal cancer. Our aim was to retrospectively study the effects of the imaging protocol on the staging accuracy.

**Patients and methods:**

MR-examinations of 37 patients with locally advanced disease were divided into two groups; compliant and noncompliant, based on the imaging protocol, without knowledge of the histopathological results. A compliant rectal cancer imaging protocol was defined as including T2-weighted imaging in the sagittal and axial planes with supplementary coronal in low rectal tumors, alongside a high-resolution plane perpendicular to the rectum at the level of the primary tumor. Protocols not complying with these criteria were defined as noncompliant. Histopathological results were used as gold standard.

**Results:**

Compliant rectal imaging protocols showed significantly better correlation with histopathological results regarding assessment of anterior organ involvement (sensitivity and specificity rates in compliant group were 86% and 94%, respectively vs. 50% and 33% in the noncompliant group). Compliant imaging protocols also used statistically significantly smaller voxel sizes and fewer number of MR sequences than the noncompliant protocols

**Conclusion:**

Appropriate MR imaging protocols enable more accurate local staging of locally advanced rectal tumors with less number of sequences and without intravenous gadolinium contrast agents.

## Background

Total mesorectal excision (TME) is the standard surgical treatment used for patients with primary rectal cancer. TME involves removal of a distinct anatomic compartment, the mesorectum, containing the rectal tumor, all local draining nodes and the mesorectal fat, by means of sharp dissection along the mesorectal fascia [1-3]. There is substantial evidence for efficacy of neoadjuvant therapy in combination with TME as being important to reduce local tumor recurrence rates [4-7]. When performing TME, knowledge of the relationship of the tumor to the circumferential resection margin (CRM) is of importance. When CRM is involved by the tumor, the risk of local recurrence is high [8-16]. The local prognostic factors assessed at preoperative magnetic resonance imaging (MRI) of rectal cancer include the extent of extramural tumor spread, involvement of the lateral resection margin, involvement of neighboring organs in the pelvis, presence of local lymph node metastases, extramural lymphovascular infiltration and peritoneal involvement [15,17]. This information helps select patients who should receive neoadjuvant treatment. This applies especially to cases with locally advanced rectal cancer, in order to maximize the chances of a complete resection and survival [18,19], and at the same time, to minimize morbidity and loss of quality of life. It is therefore of paramount interest to provide detailed anatomic knowledge of tumor and tumor invasion toward neighboring organs before treatment.

Although evaluated in several studies during the past two decades, it is only during recent years that MRI gained wide acceptance as a valuable method for assessment in patients with rectal cancer [20-33].

As a tertiary referral center responsible for patients with advanced rectal cancer, we assess magnetic resonance (MR) examinations from other institutions and hospitals at multidisciplinary team (MDT) meetings. When demonstrating these examinations at MDT meetings, variations in imaging sequences among different centers are noted. These differences may be related to both different equipments and level of dedicated experience in pelvic MRI.

To our knowledge, no study has reported the importance of the imaging protocol for assessment of tumor involvement of neighboring organs in locally advanced rectal cancer. The aim of the present study was to compare the equivalence between MRI and histopathology in patients with locally advanced rectal cancer based on the effects of using different MRI protocols.

## Patients and methods

Forty-one patients assessed as clinically suspicious for locally advanced primary rectal cancer by surgeons from 2000 to 2005, were included. 37 patients, 27 male and 10 female, with a mean age of 60.1 ± 9.8 (mean ± SD, range 28–79) who had available MRI of the pelvis were studied further. The surgeon's decision that a cancer might be advanced was based on findings at diagnostic laparotomy and/or by means of digital rectal examination.

### Radiological assessment

All examinations were provided from ten different hospitals or institutions (two of which were university hospitals). Each MR examination (all done on 1.5 T) was assessed by two or three radiologists (C.T., M.R.T. and L.B.) in consensus without knowledge of the clinical and histopathological results prior to this study according to a standard evaluation looking specifically at which organs and/or structures had been involved. However, the radiologists were aware of the high suspicion for locally advanced tumors by the clinicians. Radiologists had evaluated the morphological characteristics of the primary tumor, local prognostic factors including threatening or involvement of the mesorectal fascia, and adjacent organs in each patient.

For the part of this study, anterior organs were defined as those positioned ventral to the rectum and included the seminal vesicles, the prostate gland, the perineal body, uterus, vagina, ovaries, the small and large intestines, and the urinary bladder. Inferior and posterior organs had been defined as those that were located inferior and dorsal to the rectum, respectively, and included the levator ani muscles, obturator muscles, piriformis muscles and the sacral bone. Involvement of the abovementioned organs was defined as T4-tumor stage.

The imaging protocol of each MR-examination was recorded by one author (C.T.). Those examinations that showed the following prerequisites were defined as compliant rectal imaging protocol vs. those that did not demonstrate the same sequences (called henceforth noncompliant):

1. Sagittal and axial T2-weighted images of the pelvis performed,

2. T2-weighted images with equal to or less than 3 mm slice thickness perpendicular to the rectal length at the level of the tumor with a 16–20 cm field of view and at least a 256 × 256 matrix, otherwise called 'high resolution imaging' [20,21,25,34].

3. For low rectal tumors, coronal imaging obtained.

If the patients underwent MR examinations twice but at two different institutions, with different protocols, one compliant and the other non compliant; these were noted separately as combination protocol but categorized with the compliant group regarding some aspects. The number of other sequences and different types of artifacts (if distinguishable) were also noted.

The common denominators of all MR examinations, whether compliant or otherwise, were that they had to be performed on the request of a surgeon or oncologist for assessment of local extension of the rectal tumor preoperatively, and that the radiologist at the primary institution had not called the examination incomplete.

### Histopathological examination

All evaluations were performed according to the protocol of Quirke, et al [16,35], by one pathologist (J.L.) with more than 10 years of experience in gastrointestinal pathology. The pathologist was blinded to the MRI study protocol. The tumor site was sliced transversely at 0.5–1.0-cm intervals. The extent of tumor spread into mesorectal fascia and other structures or organs was assessed both macroscopically and with high magnification. Tumor extension into the surrounding structures and organs at microscopical examination were used as the standard of reference against which MRI findings were compared. The extension of tumor cells into mesorectal fascia and other structures or organs was assessed from inspection of the histological macrosection by light microscopy at 20× – 200× magnification.

### Statistical analysis

All MRI findings including the size of tumor, the name and number of involved fascia(e) and organ(s), the pattern of tumor involvement according to MRI and histopathology as well as the MR imaging protocol were recorded using Microsoft Excel 2003 and Microsoft Access 2000. Sensitivity and specificity of MRI between different groups were compared and 95% confidence interval (CI) was calculated with P-value < 0.05 considered significant using Stat View J-5.0 (SAS Institute. Inc., Cary, NC).

### Ethical considerations

The study was approved by the local ethical committee. No separate informed consent was obtained for this retrospective study.

## Results

### Tumor staging according to MRI

Nineteen patients were evaluated as T4 rectal tumors based on MRI. The remaining 18 were evaluated as T3 tumors without obvious invasion of neighboring organ.

### Assessment of imaging quality

Eleven patients were assessed as having compliant (D) protocols and 13 patients as combination protocols (C) and 13 patients a noncompliant imaging (N).

Regarding imaging parameters, compliant imaging protocols were used with smaller field of view (FOV) (D, 201.7 ± 77.0 mm; N, 263.5 ± 129.8 mm; mean ± SD, p = 0.03), thinner slice thickness (D, 3.8 ± 1.4 mm; N, 5.3 ± 1.9 mm; mean ± SD, p < 0.01), smaller slice gap (D, 0.2 ± 0.9 mm; N 2.0 ± 2.4 mm; mean ± SD, p < 0.01) and smaller voxel size (D, 1.3 ± 1.5 mm^3^; N, 6.7 ± 6.0 mm^3^; mean ± SD, p < 0.01). The total number of MR sequences performed in each patient was also larger in the N group (N, 9.2 ± 3.2 sequences vs. D, 5.2 ± 0.7 sequences; mean ± SD, p < 0.01 (table 1). One patient from the noncompliant group had some motion artifacts.

**Table 1:** Comparison of various MR imaging parameters, average number of sequences in each group and imaging protocols.

	**Compliant protocol (D)**	**Noncompliant protocol (N)**	***P*-value**
Parameters on T2-WI*			
Field of view			
Mean ± SD (mm)	201.7 ± 77.0	263.5 ± 129.8	0.03
Slice thickness			
Mean ± SD (mm)	3.8 ± 1.4	5.3 ± 1.9	< 0.01
Gap			
Mean ± SD (mm)	0.2 ± 0.9	2.0 ± 2.4	< 0.01
Matrix size			
Mean (mm × mm)	0.5 × 0.5	0.9 × 1.1	0.02
Voxel size			
Mean ± SD (mm^3^)	1.3 ± 1.5	6.7 ± 6.0	< 0.01
No. of sequence			
Mean ± SD (mm)	5.2 ± 0.7	9.2 ± 3.2	< 0.01

*T2 weighted image;

### Involvement of the anterior organs

**Figure 1:**
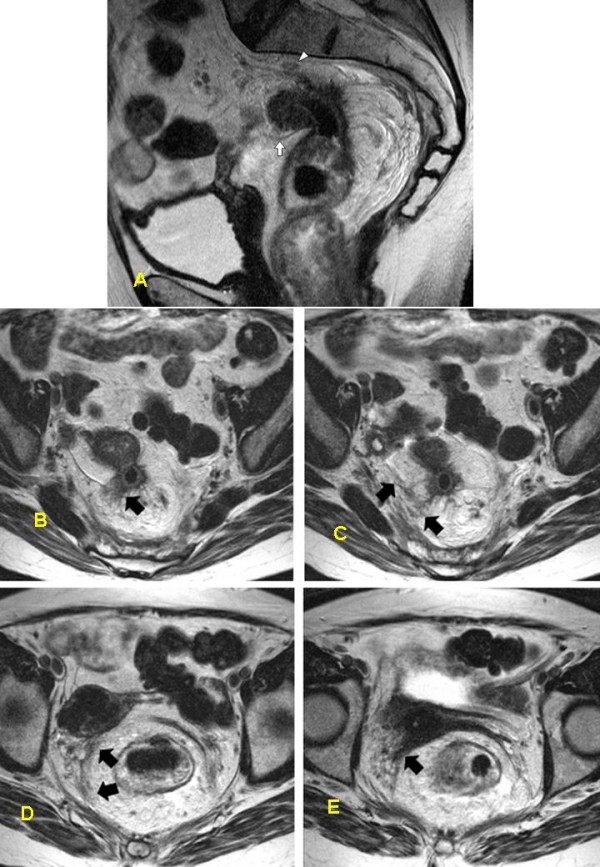
**MR images of the 'false negative' case in the group with a compliant protocol**. A-63-year-old female with rectal cancer involving the mesorectal fascia, peritoneal reflection and the parietal pelvic fascia. Imaging parameters: TR; 4056, TE; 130, NEX; 2, Thickness; 5 mm, Gap; 0 mm, FOV; 240 mm. (a) Sagittal T2-w image of the pelvis. Primary lesion is located at the rectosigmoid junction with an extramural component, extending dorsally toward the presacral fascia (arrowhead). The tumor seems to be very distant from the inner genitalia (arrow). b-e) Axial T2-w images demonstrated in a craniocaudal direction with b being the uppermost image. In b, the extramural component reaches and thickens the peritoneal fold (arrow), and more inferiorly even the pelvic side wall fascia (arrowheads in c). This fascial thickening continues (arrowheads in d, 15 mm below b), until it sweeps forward (arrow in e, 25 mm below b) and at this point the inner genitalia were involved. At the first glance, there appears to be no continuity between the tumor and the mesorectal fascia, however, histopathological examination proved tumor cells inside the fibrotic tissue and infiltrating the uterine parenchyma and the left adenxa (arrowhead in e).

In the group with compliant protocols and the group with combination protocol, preoperative MRI indicated tumor involvement of anterior pelvic organs in seven out of the 24 patients. Compared to pathological examination, six cases were true positives and one was false positive. Among the remaining 17 patients without organ involvement on MRI, pathological examination revealed one false negative case and 16 true negatives (table 2). Figure 1 demonstrates the false-negative case. In this case, there appears to be no continuity between the tumor and the uterus. However, histopathological examination showed tumor invasion along the fascia, reaching the posterior wall of the uterus and the left adnexa. The radiologist failed to ascertain the anterior extension of the tumor correctly.

**Table 2:** Comparison of various MR protocols in terms of diagnostic accuracies regarding involvement anterior to rectum.

	**Compliant and** **combination** ** protocol (D and C)**	**Noncompliant protocol (N)**
**Imaging accuracies**		
True positive	6	2
True negative	16	3
False positive	1	6
False negative	1	2
Sensitivity (%) (95% CI)	85.7 (42–99)	50.0 (6–93)
Specificity (%) (95% CI)	94.1 (71–99)	33.3 (7–70)
Positive Predictive Value (%) (95% CI)	85.7 (42–99)	25.0 (3–65)
Negative Predictive Value (%) (95% CI)	94.1 (71–99)	60.0 (14–94)

**Figure 2:**
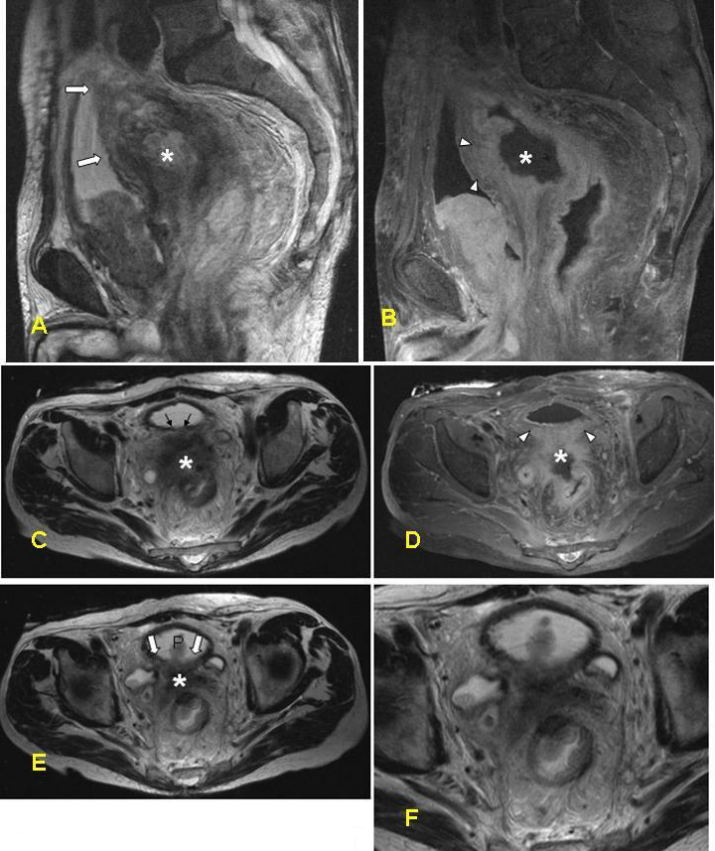
**MRI of the false positive case in the group with a noncompliant protocol**. A 76-year-old male with rectal cancer suspected of invasion to the urinary bladder. Imaging parameters: TR 7000; TE 132; NEX 2; thickness 5 mm; gap 1.5 mm; FOV 400 mm. (a) Sagittal T2-WI of the pelvis. The large primary lesion (asterisk) originating from the upper part of rectum with accompanying desmoplastic and edematous changes seems to be invading the muscular wall of the bladder dorsally (white arrows). The tumor appears to penetrate into the muscular layer of the urinary bladder which shows higher signal intensity compared to the normal part. (b) Sagittal contrast-enhanced T1-WI of the pelvis with fat-suppression. The posterior bladder wall is not distinguishable, yet the tumor is seen enriching ventrally (white arrowheads) and therefore, it is suspicious for penetrating into the bladder wall. (c-f) Corresponding axial images. c, e, and f are T2-WI and d is T1WI with contrast-enhancement and fat-suppression. T1-w images after Gadolinium contrast enhancement with fat saturation give the impression of the tumor (asterisk) growing into the dorsal wall of the urinary bladder (arrowheads). However, histopathological examination revealed no tumor involvement of the urinary bladder.

In the noncompliant imaging group, preoperative MRI was indicative of organ involvement in eight cases. Pathological examination revealed two as true positives and six as false positives (Figure 2). Among the remaining five patients without organ involvement, pathological examination revealed two false negatives and three true negatives.

Sensitivity, specificity, positive predictive value (PPV) and negative predictive value (NPV) in the compliant and combination protocol group were 85.7%, 94.1%, 85.7%, and 94.1%, respectively. On the other hand, in the group with non-compliant protocol, the sensitivity, specificity, PPV and NPV were 50.0%, 33.3%, 25.0%, and 60%, respectively. Statistically significant difference (p < 0.05) was observed regarding measured specificity (95% CI; 7–70 for group N vs. 95% CI; 71–99 for the other two groups, D and C). The difference in sensitivity in the two groups did not reach statistical significance levels (Table 2).

### Posterior or inferior organ involvement

Only three out of the present 19 patients with locally advanced tumor, showed involvement of an inferior organ (levator ani muscle, piriformis muscle) or a posterior organ (Os sacrum) by the tumor, without simultaneous involvement of any anterior organ. Two of these patients used compliant imaging, and pathological examination revealed both to be true positives. In one patient with noncompliant imaging an inferior organ involvement was suspected but pathological examination proved no obvious tumor infiltration or fibrosis in that organ (false-positive). The number of cases was too few to make any meaningful statistical analysis.

## Discussion

The results of this study indicate considerable differences in correlation between preoperative imaging and histopathology depending on the imaging protocol. Using compliant imaging, despite fewer imaging sequences, a considerably better prediction of tumor invasion towards anterior pelvic organs is seen. On the contrary, this study also indicates that MRI performed with noncompliant imaging protocol does not allow accurate prediction. One other observation is that the radiologist tends to over-stage when the imaging protocol is not optimal. This could be due to the fear of positive resection margins caused by a false negative assessment and partial volume effect observed with thick slices not obtained in the appropriate planes. This could of course be due to nature of the study as well. The radiologists assessing the MR exams were aware of the selection criteria and might have felt compelled to over-stage.

The lack of compliant imaging, and as we suspect the lack of high resolution T2-weighted imaging, probably forced the radiologists to rely on images with considerable volume averaging. Compared to the compliant imaging, both slice thickness including gap and voxel size were significantly larger in the noncompliant imaging group (*P *< 0.05). Larger slice thickness and gap yield more partial volume effect, thus leading the radiologists to make overestimation of tumor extent. In areas of the pelvis where there are small interfaces between tissues, such as in the anterior and low part of the rectum, this is probably of particular importance. In the compliant and combination groups, there was one false positive and one false negative finding of anterior organ involvement out of 24 cases.

In the noncompliant imaging group, there were six false positive and two false negative cases out of 13 cases. This means that one patient out of 24 from D and C groups and six patients out of 13 from the N group might receive unnecessary extensive surgery and prolonged, preoperative chemoradiotherapy. Anterior pelvic organs are closely related to urinary and sexual function, and anterior organ surgery has great impact on the patient's quality of life after surgery. By contrast at least partially because of false negative assessments by radiologists, one out of 24 cases from D and C groups, and two out of 13 cases from the N group had involved resection margins.

Although the low number of cases prohibits any meaningful analysis to be done regarding accuracy of MRI for assessment of organs inferior or dorsal to rectum, our findings suggest that compliant imaging might be superior to noncompliant imaging also for these patients. This low frequency could be due to less likelihood of involvement of posterior organs compared to anterior organs due to more distance between rectum and these neighboring organs [36].

The number of MR sequences was different between various groups with larger numbers observed in the noncompliant imaging group. It seems that whenever the compliant sequences were not employed, there was a tendency to conduct several other sequences. One of the most widely used sequences in the N group was the one with usage of gadolinium intravenous contrast. Recently, Vliegen and others have shown that gadolinium-enhanced MRI does not improve the diagnostic accuracy in local staging of rectal cancer [37]. Unnecessary use of contrast agents might only lead to increased rate of adverse events and increased costs and time needed for examination, without any proven benefit for the patients.

There are a number of other limitations in this study. First, we did not compare the same patients using different imaging protocols.

Second, there was a difference in the sensitivity of MR examinations using different protocols when assessing detection of anterior organ involvement, however, the difference did not reach statistical significance which is probably due to the low power of the study and perhaps the nature of the study (i.e. the radiologists knew that these cases were more likely to be advanced cases).

However, even with these limitations, the compliant imaging improves accuracy, especially in advanced and complicated cases. It is therefore of utmost importance that radiologists are made aware of pitfalls and the problems, and that radiologist are made up-to-date about recent developments in imaging. This current study reveals that there is a need for continued education in this field.

## Conclusion

For local staging of locally advanced rectal cancer, the correlation between MRI and histopathology was better when a predefined compliant rectal imaging protocol was used. It is possible that this also holds true for all patients assessed with rectal cancer and not only for anterior structures in the pelvis. However, this has to be assessed in further studies. Furthermore, this study indicates that continuous training of radiologists and radiology technicians, including work-shops and seminars seems to be an appropriate way to improve accuracy of MRI in patients with rectal cancer.

## Abbreviations

MR(I): Magnetic resonance (imaging); TME: Total mesorectal excision; CRM: Circumferential resection margin; T2-w (image): T2 weighted (image); FOV: Field of view; MDT: Multidisciplinary team; PPV: Positive predictive value; NPV: Negative predictive value; TR: Repetition Time; TE: Echo Time; NEX: number of excitations.

## Competing interests

The authors declare that they have no competing interests.

## Authors' contributions

CS idea, data collection, radiological assessment, manuscript preparation. MT idea, data collection, radiological assessment, manuscript preparation. ST idea, data collection, surgical and clinical assessment, histopathological evaluation, manuscript preparation. GP idea, data collection, surgical and clinical assessment, manuscript preparation. TH idea, data collection, surgical and clinical assessment, histopathological evaluation, manuscript preparation. JL idea, data collection, histopathological evaluation, manuscript preparation. LB idea, supervision, manuscript preparation. All authors read and approved the final version
